# Examining the Relationship Between Schizotypy and Self-Reported Visual Imagery Vividness in Grapheme-Color Synaesthesia

**DOI:** 10.3389/fpsyg.2016.00131

**Published:** 2016-02-29

**Authors:** Agnieszka B. Janik McErlean, Michael J. Banissy

**Affiliations:** ^1^Department of Psychology, Goldsmiths University of LondonLondon, UK; ^2^Department of Psychology, James Cook UniversitySingapore, Singapore

**Keywords:** synaesthesia, schizotypy, imagery, sensation seeking, self-monitoring

## Abstract

Synaesthesia is a condition in which one property of a stimulus triggers a secondary experience not typically associated with the first (e.g., seeing achromatic graphemes can evoke the perception of color). Recent work has explored a variety of cognitive and perceptual traits associated with synaesthesia. One example is in the domain of personality, where higher rates of positive schizotypy and openness to experience and lower agreeableness have been reported in synaesthetes who experience color as their evoked sensation relative to typical adult controls. Additionally, grapheme-color synaesthetes have previously been reported to show elevated mental imagery compared to typical adults. Here, we aimed to further elucidate the relationship between personality, synaesthesia, and other cognitive traits. In Study 1, we examined self-reported schizotypy and self-reported visual imagery vividness in grapheme-color synaesthetes and typical adults. Our results partially replicated previous findings by showing that synaesthesia was associated with greater positive schizotypy and enhanced self-reported imagery vividness. The results also extend previous reports by demonstrating that differences in positive schizotypy and mental imagery vividness are not related in grapheme-color synaesthesia. In Study 2, we sought to build on prior work showing lower agreeableness and increased openness to experience in synaesthetes by examining whether grapheme-color synaesthesia is associated with other conceptually related traits; namely lower self-monitoring and increased sensation seeking. We did not find any differences between synaesthetes and controls on either of these traits. These findings are discussed in relation to potential factors that may contribute to the observed personality profile in grapheme-color synaesthesia.

## Introduction

Synaesthesia is a condition in which stimulation in one modality leads to involuntary secondary sensations within the same or different modality (Sagiv, 2004). For example, in grapheme-color synaesthesia perceiving letters or numbers printed in black ink triggers secondary color experiences (e.g., the letter A is perceived as red or the number 8 as green). Many types of synaesthesia have been identified including time-space synaesthesia (days and months are visualized in specific spatial arrangements; Simner et al., 2009), synaesthesia involving touch (Ward et al., 2008a), and lexical-gustatory synaesthesia (spoken words induce taste sensations; Ward and Simner, 2003). The authenticity of the condition is well established and there has been growing interest in using synaesthetes as a unique experimental population to investigate wider aspects of perception and cognition that extend beyond the synaesthetic experience itself. For instance, recent work has demonstrated an association between synaesthesia and enhanced memory (Rothen et al., 2012), sensory perception (Yaro and Ward, 2007; Banissy et al., 2009, 2013b), and creativity (Ward et al., 2008b).

There is also some evidence suggesting that synaesthesia might be linked to a specific personality profile. For example, color synaesthesia has been recently associated with positive and disorganized schizotypy (Banissy et al., 2012). Schizotypy is a sub-clinical construct tapping normative dispositions toward characteristics that are associated with schizophrenia (Mason and Claridge, 2006). Non-synaesthetic artistic individuals have also been found to score high on positive schizotypy (Nelson and Rawlings, 2010), which is interesting in the context of reports showing that synaesthetes themselves tend to gravitate toward creative industries (Ward et al., 2008b) and art-related university courses (Rothen and Meier, 2010). There is also some evidence suggesting that compared to controls, synaesthetes score higher on creativity measures (Ward et al., 2008b) and show enhanced self-reported imagery vividness (Barnett and Newell, 2008; Spiller et al., 2015). These skills have also been previously linked to schizotypy in the general population (Oertel et al., 2009). With this in mind, one account for why synaesthetes show heightened levels of positive and disorganized schizotypy may be because these traits are part of broader constellation of characteristics (e.g., increased creativity and mental imagery) associated with synaesthesia (Banissy et al., 2012). An alternative possibility is that previously reported differences in schizotypy in synaesthesia for color reflect some degree of similarity in mechanisms that might contribute to synaesthesia and schizotypy. For example, synaesthesia and schizophrenia have both been linked to abnormalities in early stages of visual processing (Butler and Javitt, 2005; Barnett et al., 2008; Banissy et al., 2013b). In schizophrenia, these deficits have been associated with NMDAR hypofunction (Butler and Javitt, 2005) and it is feasible that similar signaling deficits may be related to reductions of cortical inhibition in synaesthesia (e.g., Brang and Ramachandran, 2008). A direct examination of the relationship between differences in schizotypy and mental imagery in synaesthesia for color is lacking, thus investigating this relationship further is important to help constrain our understanding of factors that contribute to synaesthesia.

Synaesthetes who experience color as their concurrent (i.e., their evoked sensation) have also been found to score higher on the openness to experience, and lower on agreeableness subscales of the Big Five Inventory (John et al., 1991; Banissy et al., 2013a). These synaesthetes also showed higher scores on the fantasizing dimension of the Interpersonal Reactivity Index (Davis, 1980; Banissy et al., 2013a). Again, a similar personality profile has previously been reported in visual artists (Burch et al., 2006). This finding is consistent with the aforementioned reports of a higher prevalence of synaesthetes in creative professions (Ward et al., 2008b) and the possibility that there may be a constellation of personality traits associated with synaesthesia.

In the general population, a number of other traits have been associated with personality dimensions that have been shown to be different in color synaesthetes (e.g., openness to experience, agreeableness). For example, sensation seeking and openness to experience both relate to the desire to seek novel and exciting sensations and experiences. García et al. (2005) found sensation seeking (in particular experience seeking) to be a good predictor of openness to experience. Sensation seeking has also been found to be positively linked to the novelty seeking subscale of Temperament and Character Inventory (Cloninger, 1994), which has been positively associated with the Five Factor Model trait of openness to experience and inversely associated with conscientiousness (De Fruyt et al., 2000). Further, self-monitoring (SM) is a personality trait that has been suggested to relate to the agreeableness subscale of the Big Five Inventory (Keller, 1999). This trait reflects the degree to which a person regulates his/her behavior in order to adapt to social situations. People who score high on this measure tend to closely monitor their behavior and to be more responsive to various social cues. On the other hand, low self-monitors tend to be more expressive of their own opinions and feelings and less adaptable in a social context. As yet, the question of whether levels of SM or sensation seeking differ in synaesthetes relative to the general population has not been examined.

With this in mind, the primary objectives of the current study were as follows. Firstly, we aimed to replicate previous findings linking synaesthesia for color with enhanced self-reported positive and disorganized schizotypy (Banissy et al., 2012), and mental imagery vividness (Barnett and Newell, 2008; Spiller et al., 2015). Secondly, we investigated whether greater self-reported imagery vividness present in synaesthesia for color mediates elevated levels of schizotypy in this group. Finally, based on previous findings showing lower agreeableness and increased openness to experience in synaesthetes (Banissy et al., 2013a) we aimed to establish if synaesthesia for color would be associated with other conceptually similar personality characteristics. Specifically, we hypothesized that synaesthesia might be linked to lower SM and increased sensation seeking. To do so we conducted two studies, with Study 1 focusing on mental imagery and schizotypy in synaesthesia, and Study 2 investigating SM and sensation seeking in synaesthesia.

## Study 1: Imagery and Schizotypy

### Method

#### Participants

Thirty-five grapheme-color synaesthetes (32 female, three male age *M* = 38.6 *SD* = 15.05) and 35 age and gender matched controls (32 female, three male age *M* = 38.51 *SD* = 17.07) took part in this study. Some of the synaesthetes also reported spatial forms (7), personification (6), mirror-touch (3), and other forms of synaesthesia involving color (16). They were recruited from a database of verified synaesthetes. We invited 110 synaesthetes to take part, 11 of whom had previously taken part in the study by Banissy et al. (2012). To preserve anonymity the participants did not provide personal identifying details when completing the questionnaires online. Therefore we cannot trace exactly which synaesthetes from our invited sample took part in the study. All synaesthetes had been previously tested either using the online Eagleman Synaesthesia Test Battery (Eagleman et al., 2007) where a score below 1 indicates a presence of grapheme-color synaesthesia (also see Rothen et al., 2013), or using a test-retest consistency over time method (all synaesthetes showing > 85% consistency over time). The controls were recruited from the student population and via acquaintances. They were asked whether they had been previously verified as synaesthetes and if not whether they suspected they might have grapheme-color synaesthesia (which was explained as a condition where one experiences letters or numbers in particular colors), or whether they thought they might have other forms of this condition. They were also provided with a link to the Eagleman’s test which they were asked to do if they suspected they might have synaesthesia. Those who were verified as synaesthetes were excluded from the control group. Participants were entered into a prize-draw competition (£50 shopping voucher) as compensation for their time. All participants gave informed consent and the study was approved by the local ethics committee of Goldsmiths (University of London).

#### Materials

Participants completed two measures online. One was the Oxford-Liverpool Inventory of Feelings and Experiences (O-Life, Mason and Claridge, 2006). This standardized instrument is designed to measure schizotypal traits in non-clinical groups and has been used previously in the study of synaesthetes by Banissy et al. (2012). It consists of 104 items and has four subscales: UEs (30 items), Introvertive Anhedonia (27 items), Cognitive Disorganization (24 items) and Compulsive Non-Conformity (23 items). This measure has a dichotomous response format where ‘yes’ is scored as 1 and ‘no’ as 0 accept for negatively worded items.

Additionally all subjects completed the Vividness of Visual Imagery Questionnaire VVIQ (Marks, 1973), which is a widely used measure of self-reported imagery and was used in previous studies examining mental imagery in synaesthesia (Barnett and Newell, 2008; Spiller et al., 2015). Participants were required to rate their imagery on a five point scale (1 – perfectly clear and as vivid as normal vision, 2 – clear and reasonably vivid, 3 – moderately clear and vivid, 4 – vague and dim, 5 – no image at all, only ‘knowing’ that you are thinking of the object) with eyes open, then with eyes closed, resulting in two separate scores (maximum 80) which when summed together give a total VVIQ score (maximum 160). The lower the score on this measure the greater the vividness of visual imagery.

### Results

**FIGURE 1:**
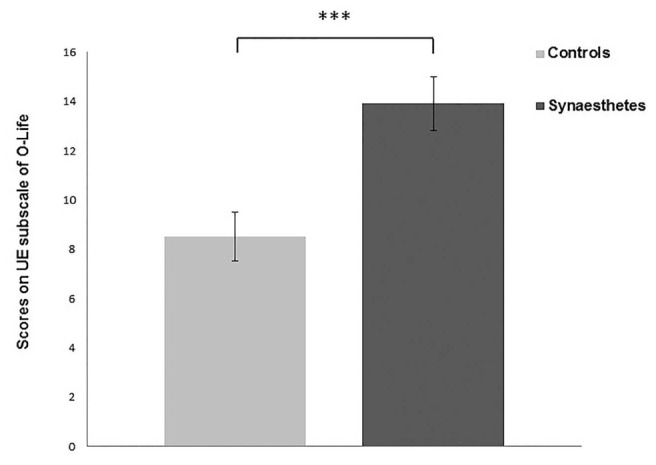
**Synaesthetes scored significantly higher on the Unusual Experiences (UEs) subscale of O-Life indicating greater positive schizotypy in this group.** Error bars show SEM. ^∗∗∗^*p* < 0.001.

**FIGURE 2:**
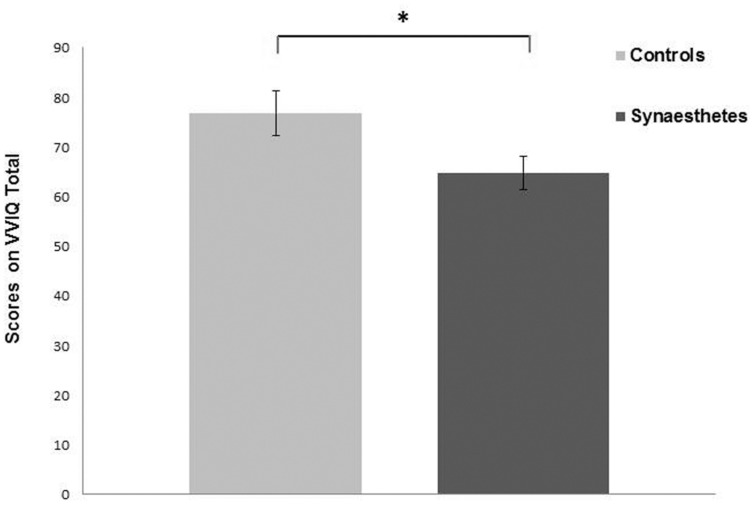
**Synaesthetes had a lower VVIQ Total score compared to controls indicating greater self-reported imagery in this group.** Error bars show SEM. ^∗^*p* < 0.05.

Only the UEs subscale of O-Life and Total VVIQ (TVVIQ) scale were normally distributed. Both UE and TVVIQ had high consistency scores with Cronbach’s Alpha of 0.884 and 0.972, respectively. Both UE and TVVIQ were then analyzed using separate independent samples *t*-tests. Synaesthetes scored higher on UE subscale compared to controls [*t*(68) = 3.697, *p* = <0.001, Cohen’s *d* = 0.88] (**Figure 1**), indicating higher levels of positive schizotypy in synaesthetes; therefore replicating one aspect of previous findings (Banissy et al., 2012). Synaesthetes also reported significantly greater self-reported visual imagery than controls [*t*(68) = 2.160, *p* = 0.034, Cohen’s *d* = 0.51] replicating previous findings (Barnett and Newell, 2008; Spiller et al., 2015) (**Figure 2**).

All other subscales, which were not normally distributed, were analyzed with non-parametric independent-samples Mann–Whitney *U* tests. The only significant difference between synaesthetes and controls was on the VVIQ open eyes subscale of VVIQ measure [*U*(70) = 389.500, *z* = -2.622, *p* = 0.009, *r* = -0.31] where synaesthetes reported greater imagery than controls. There were no other statistically significant differences (Introvertive Anhedonia: *U*(70) = 651.500, *z* = 0.460, *p* = 0.646, *r* = 0.05; Cognitive Dizorganization: *U*(70) = 692.000, *z* = 0.935, *p* = 0.350, *r* = 0.01; Compulsive Non-Conformity: *U*(70) = 674.00, *z* = 0.726, *p* = 0.468, *r* = 0.08; VVIQ closed eyes: *U*(70) = 497.50, *z* = -1.352, *p* = 0.177, *r* = -0.16).

#### The Relationship Between Elevated Schizotypy and Visual Imagery in Synaesthesia

To examine the relationship between elevated schizotypy and visual imagery scores in synaesthetes, we also calculated Pearson Correlation Coefficients. The correlation between TVVIQ and UE was not significant in either synaesthetes [*r*(35) = -0.169, *p* = 0.330] or controls [*r*(35) = -0.081, *p* = 0.644].

**FIGURE 3:**
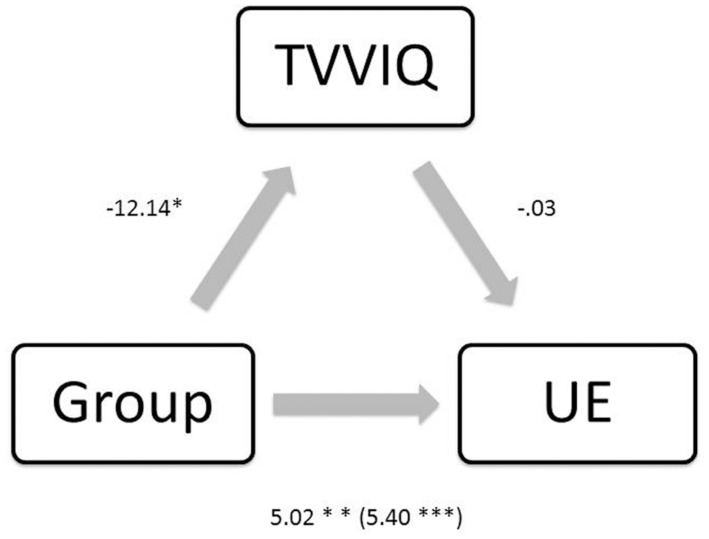
**Regression coefficients for the relationship between group and UE (schizotypy) are not mediated by TVVIQ (imagery).** ^∗^*p* < 0.05, ^∗∗^*p* < 0.01, ^∗∗∗^*p* < 0.001.

Additionally, we ran a mediation analysis using bootstrapping with bias-corrected confidence estimates (Hayes, 2013) to assess the interaction between schizotypy, visual imagery, and synaesthesia. Ninety-five percent confidence interval of the indirect effect was obtained with 1000 bootstrap samples. As **Figure 3** shows group membership (being a synaesthete as opposed to a control participant) was positively associated with schizotypy [*B* = 5.40, *t*(68) = 3.69, *p* < 0.001] and negatively associated with imagery (as lower scores on TVVIQ indicate grater vividness of visual imagery; *B* = -12.14, *t*(68) = -2.15, *p* = 0.034]. The results also showed that the association between imagery and schizotypy was not significant (*B* = -0.03, *t*(67) = -0.98, *p* = 0.330]. The mediation analysis did not show a mediating role of imagery on the relationship between group and schizotypy [*B* = 3.54, CI (-0.19, 1.73), *Z* = 0.82, *p* = 0.410, *K*^2^ = 0.03]; model statistics between group and schizotypy when controlling for imagery were *B* = 5.02, *t*(67) = 3.32, *p* = 0.001. In this regard, elevated self-reported visual imagery did not mediate the relationship between increased positive schizotypy and presence of synaesthesia for color.

**FIGURE 4:**
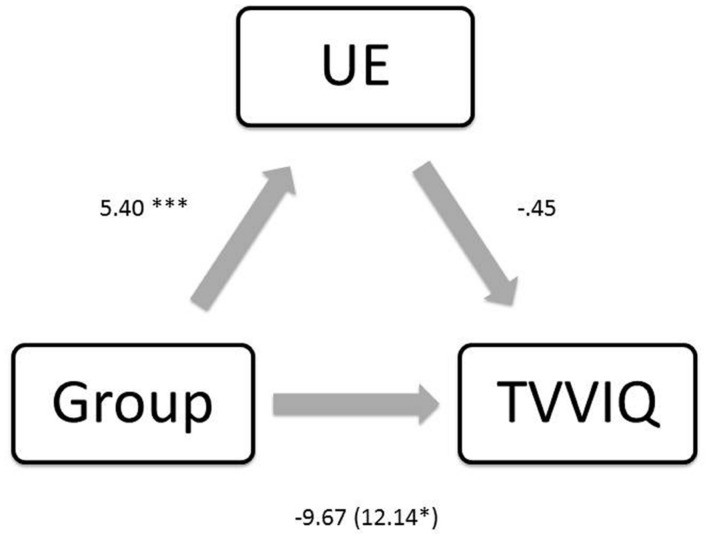
**Regression coefficients for the relationship between group and TVVIQ (imagery) are not mediated by UE (schizotypy).** ^∗^*p* < 0.05, ^∗∗∗^*p* < 0.001.

An additional mediation analysis was run in order to determine whether the presence of schizotypy would mediate levels of imagery using the same method as above (**Figure 4**). Again, there was a negative association between group and TVVIQ [*B* = -12.14, *t*(68) = -2.15, *p* = 0.034], and a positive association between group and schizotypy [*B* = 5.40, *t*(68) = 3.69, *p* = <0.001]. There was no significant association between schizotypy and imagery [*B* = -0.45, *t*(67) = -0.98, *p* = 0.330]. Although the significance level of the relationship between group and imagery was reduced when controlling for schizotypy [*B* = -9.67, *t*(67) = -1.568, *p* = 0.121], the mediation effect was not significant suggesting that schizotypy is not a significant mediator of the relationship between group and imagery [*B* = -2.47, CI (-8.80, 2.19); *Z* = -0.91, *p* = 0.359, *K*^2^ = 0.04].

## Study 2: Sensation Seeking and Self-Monitoring

Taking into account recent findings demonstrating that synaesthesia for color is linked to lower agreeableness as well as increased openness to experience subscales of the Big Five Inventory (Banissy et al., 2013a) we sought to determine whether this form of synaesthesia would also be associated with other conceptually related personality characteristics; namely lower SM and increased sensation seeking.

### Material and Methods

#### Participants

The same sample of synaesthetes that were invited to take part in Study 1 (i.e., *n* = 110 synaesthetes) were invited to take part in this study. From this 37 grapheme-color synaesthetes (34 female, three male age *M* = 41.27 *SD* = 17.58) and 37 age and gender matched controls (34 female, three male age *M* = 38.70 *SD* = 17.82) took part. Some of the synaesthetes also reported spatial forms (4), personification (3), and mirror-touch (2) synaesthesia, and other forms of synaesthesia involving color (9). For all synaesthetic participants, synaesthesia had been confirmed either using an online consistency test (Eagleman et al., 2007), or using a test–retest method carried out on two separate occasions. The controls were recruited from the student population via Mahara (an online platform used for advertising experiments) and via acquaintances. Participants were screened for synaesthesia and entered into a prize-draw competition (£50 shopping voucher) as payment for their time. All participants gave informed consent and the study was approved by the local ethics committee of Goldsmiths (University of London).

#### Materials

Participants completed two measures online. One was the Sensation Seeking Scale (SSS-V; Zuckerman, 1979, 1995), which measures a person’s need to experience new and stimulating experiences and sensations. This widely used instrument has a dichotomous response format where participants must choose one of two statements which best describes their likes and feelings (e.g., A: ‘I get bored seeing the same old faces’, B: ‘I like the comfortable familiarity of everyday friends’). SSS-V has four underlying dimensions each containing 10 items: thrill and adventure seeking (TAS), experience seeking (ES), disinhibition (D), and boredom susceptibility (BS), which summed together give a total sensation seeking score (maximum 40).

Participants also completed the SM scale (Snyder and Gangestad, 1986). SM is a personality trait whereby a person adapts his/her behavior in order to fit into a social context. Participants who score high are classified as high self-monitors. These individuals tend to be concerned about their social desirability and thus regulate their behavior in order to adapt to the social situations. On the other hand, low self-monitors are less concerned with the judgment or opinion of others, are more expressive of their own views and tend to be less socially adaptable (Friedman and Miller-Herringer, 1991; Flynn et al., 2006). Self-Monitoring scale (Snyder and Gangestad, 1986) consists of 18 statements such as, e.g., ‘I would not change my opinions (or the way I do things) in order to please someone or win their favor’. Participants need to indicate if the statements in relation to their own feelings and experiences are true or false. Each item is keyed such that high-self monitors tend to indicate answers in the keyed direction, while low-self monitors tend to opt for the alternative answer. The maximum possible score on this measure is 18.

### Results

Only the SM scale was normally distributed. Cronbach’s alpha for this measure was 0.771. Independent samples *t*-test revealed that the difference in SM between synaesthetes and controls was not significant [*t*(61,116) = -1.268, *p* = 0.209, Cohen’s *d* = -0.29]. Similarly, no significant differences were found between the groups on the Sensation Seeking subscales using Independent-Samples Mann–Whitney U tests [TAS: *U*(74) = 677, *p* = 0.935, *r* = < 0.001; ES: *U*(74) = 636, *p* = 0.596, *r* = 0.06; D: *U*(74) = 638, *p* = 0.617, *r* = 0.05; BS: *U*(74) = 624, *p* = 0.512, *r* = 0.07; TOTAL: *U*(74) = 623.50, *p* = 0.509, *r* = 0.07] (see **Table 1** for more statistics).

**Table 1:** Means and Standard Deviations for normally distributed individual subscales of O-Life, VVIQ, SSS-V and SM, and Medians and Ranges for subscales which were not normally distributed.

Individual subscales			
		**Mean**	***SD***
Unusual experiences (UEs)	Controls	8.51	5.80
	Synaesthetes	13.91	6.40
VVIQ total	Controls	76.82	26.58
	Synaesthetes	64.68	19.99
Self-monitoring (SM)	Controls	9.21	2.92
	Synaesthetes	10.35	4.58
		**Median**	**Range**
Cognitive disorganization	Controls	10	24
	Synaesthetes	15	21
Introvertive Anhedonia	Controls	6	17
	Synaesthetes	7	21
Compulsive non-conformity	Controls	6	14
	Synaesthetes	8	18
VVIQ eyes open	Controls	35	50
	Synaesthetes	30	40
VVIQ eyes closed	Controls	36	60
	Synaesthetes	33	47
Thrill and adventure seeking	Controls	4	10
	Synaesthetes	4	10
Experience seeking	Controls	6	9
	Synaesthetes	6	8
Disinhibition	Controls	4	10
	Synaesthetes	5	9
Boredom susceptibility	Controls	3	8
	Synaesthetes	2	7
Sensation seeking total	Controls	19	33
	Synaesthetes	17	26


## General Discussion

This study examined the relationship between increased self-reported mental imagery and schizotypy in grapheme-color synaesthesia. Building on prior work indicating that greater positive and disorganized schizotypal traits amongst individuals with synaesthesia for color (Banissy et al., 2012) and increased self-reported mental imagery vividness in synaesthesia for color (Barnett and Newell, 2008; Spiller et al., 2015) it was expected that grapheme-color synaesthetes would differ on these measures relative to controls. Further given prior suggestions that differences in schizotypy levels found between synaesthetes and controls may be related to comorbidity between schizotypy and mental imagery vividness, we sought to examine the relationship between visual imagery and schizotypy in the same sample of synaesthetes.

Our findings partially replicate Banissy et al.’s (2012) results by showing that synaesthetes who experience color as their evoked sensation score higher on positive schizotypy, but not other aspects of schizotypy. We note that we did not replicate the Banissy et al. (2012) finding of differences between synaesthetes who experience color as their evoked sensation and disorganized schizotypy. Consistent with previous findings, we also found synaesthesia for color to be linked with greater self-reported imagery vividness compared to controls (Barnett and Newell, 2008; Spiller et al., 2015). By comparing levels of self-reported mental imagery vividness and schizotypy in the same sample of synaesthetes, we were also able to determine that elevated schizotypal traits present in grapheme-color synaesthetes were not related to self-reported imagery vividness. This suggests that increased levels of these traits in grapheme-color synaesthetes may be independent of each other, and constrains explanations of why grapheme-color synaesthetes may have heightened positive schizotypy. Moreover, prior accounts have suggested that heightened levels of positive and disorganized schizotypy in synaesthesia for color may be because these traits are part of broader constellation of characteristics (e.g., increased mental imagery) that have been associated with the presence of synaesthesia (Banissy et al., 2012). That grapheme-color synaesthetes did not show a relationship between positive schizotypy and self-reported mental imagery vividness in the current study argues against this possibility. This pattern of data is also consistent with previous findings demonstrating that while non-synaesthetic individuals with increased schizotypal traits score higher on vividness of mental imagery, these two constructs appear to be independent of each other (Oertel et al., 2009). It will therefore be important for future work to determine factors contributing to elevated positive schizotypy and self-reported vividness of mental imagery in synaesthesia for color.

One possible reason for elevated positive schizotypy in synaesthesia for color may be that there is some degree of similarity in mechanisms contributing to synaesthetic, and the unusual visual experiences associated with positive schizotypy. For example, synaesthesia and schizophrenia have both been linked to abnormalities in early stages of visual processing (e.g., Butler and Javitt, 2005; Barnett et al., 2008). In schizophrenia, these deficits have been associated with NMDAR hypofunction (Butler and Javitt, 2005) and it is feasible that similar mechanisms may be related to reductions of cortical inhibition in synaesthesia (e.g., Brang and Ramachandran, 2008). Understanding factors that contribute to the relationship between elevated positive schizotypy and grapheme-color synaesthesia will therefore be an important line of future study in order to constrain our understanding of mechanisms that are linked to both synaesthesia and schizotypy. Further, it will be important for future work to extend our mediation analysis conducted here to a larger sample. Interpreting null results is always a challenge, and in the context of mediation analyses our grapheme-color synaesthete sample size was relatively modest. The results found here therefore require future replication. We note, however, that prior work in non-synaesthetic participants using the same measures and a larger sample than ours found similar results on the relationship between schizotypy and imagery in non-synaesthetes (Oertel et al., 2009; Bell and Halligan, 2010).

Additionally, as this study tested grapheme-color synaesthetes (some of whom also reported other forms of synaesthesia) on vividness of visual imagery we can only make inferences about visual forms of synaesthesia and imagery within the visual domain. Spiller et al. (2015) found that while synaesthetes tend to show heightened imagery in the modality of their synaesthetic experiences relative to controls and other synaesthetes (i.e., those that experience synaesthesia in a different modality), synaesthetes also showed a general enhancement of imagery across various modalities in which they do not experience synaesthesia relative to non-synaesthetes. In this context, one may expect some difference in visual imagery irrespective of the modality of synaesthesia, but predictions regarding differences in positive schizotypy are less clear. While theories of synaesthesia (e.g., in relation to cortical inhibition) tend to take a one size fits all approach, in practice our knowledge of factors that contribute to synaesthetic experience are largely restricted to studies of grapheme-color synaesthesia. Therefore the extent to which grapheme-color synaesthesia is a consequence of domain-general (i.e., common to all types of synaesthesia) or domain-specific (i.e., specific to grapheme-color) factors is unclear. Understanding domain-general and domain-specific factors that contribute to grapheme-color synaesthesia (and other variants of synaesthesia), and how these factors are associated with broader traits (e.g., schizotypy) will help to constrain predictions on putative relationships between different variants of synaesthesia and wider aspects of perception and cognition that extend beyond the synaesthetic experience itself.

Another question is whether the number of synaesthesia forms could influence current results. Based on prior findings suggesting an association between the number of synaesthetic modalities and vividness of reported imagery (Spiller et al., 2015) it could by hypothesized that individuals with multiple forms of synaesthesia stretching across different modalities would report elevated levels of visual imagery relative to those with fewer synaesthetic modalities. However, greater vividness of visual imagery should not influence the findings on schizotypy, as enhanced imagery and schizotypy appear to be independent of each other (Oertel et al., 2009; Bell and Halligan, 2010; current study), and thus greater intensity of imagery in individuals with different synaesthetic forms would not be expected to alter this relationship.

Based on previous findings demonstrating an association between synaesthesia and greater openness to experience (Banissy et al., 2013a), and studies showing that openness to experience and sensation seeking are conceptually related (García et al., 2005), we also investigated if grapheme-color synaesthesia would be linked to greater sensation seeking and lower SM. However, we did not find this. One reason for this might be due to methodological weaknesses of the SSS-V. For example, the SSS-V requires participants to make forced-choice answers, (e.g., between options such as A: ‘I am not interested in experience for its own sake’ or B: ‘I like to have new and exciting experiences and sensations even if they are a little frightening, unconventional or illegal’) and, although some of the items have been revised, it is still sometimes considered to be a dated measure (Gray and Wilson, 2007). In this regard, a questionnaire using a Likert-type format may have been more sensitive. That being said, prior work linking openness to experience with sensation seeking in the general population (García et al., 2005) administered the same questionnaire as used here, thus the measure can be considered reliable. A further possibility is that as synaesthesia results in very rich sensory experiences, which at times might be overwhelming, synaesthetes may not actively seek situations that would provide even more sensory stimulation.

In light of previous research showing an association between synaesthesia and decreased agreeableness (Banissy et al., 2013a), it was also hypothesized that grapheme-color synaesthesia would be linked to low SM because both constructs relate to a persons’ ability and desire to cooperate, and adjust their behavior to a social context (Keller, 1999). Nevertheless, we found no difference in behavior monitoring between synaesthetes and controls. In conjunction with the findings of Banissy et al. (2012, 2013a) and the findings from Study 1 of differences between synaesthetes and controls on specific trait dimensions (e.g., positive schizotypy, but not other traits), the lack of a difference in sensation seeking and SM in synaesthetes implies that grapheme-color synaesthesia is linked to a specific personality profile and that these differences are not due to a general self-report bias in synaesthetes.

In sum, we demonstrated that grapheme-color synaesthetes show greater positive schizotypy and self-reported visual imagery vividness compared to controls. By examining these two constructs in one sample of synaesthetes, we also found that heightened positive schizotypy is not a result of a relationship between schizotypy and mental imagery vividness. Finally, synaesthetes did not differ from controls in terms of levels of SM or sensation seeking despite previous work reporting that color synaesthetes differ from controls on agreeableness and openness to experience (Banissy et al., 2013a), suggesting a specific personality profile difference in synaesthesia for color.

## Author Contributions

AJM collected and analayzed the data. The authors contributed equally in all other aspects.

## Conflict of Interest Statement

The authors declare that the research was conducted in the absence of any commercial or financial relationships that could be construed as a potential conflict of interest.
